# Defects in Base Excision Repair Sensitize Cells to Manganese in *S. cerevisiae*

**DOI:** 10.1155/2013/295635

**Published:** 2013-10-27

**Authors:** Adrienne P. Stephenson, Tryphon K. Mazu, Jana S. Miles, Miles D. Freeman, R. Renee Reams, Hernan Flores-Rozas

**Affiliations:** Florida A&M University, College of Pharmacy & Pharmaceutical Sciences, 1520 Martin Luther King Boulevard, Dyson Building Room 221, Tallahassee, FL 32307, USA

## Abstract

Manganese (Mn) is essential for normal physiologic functioning; therefore, deficiencies and excess intake of manganese can result in disease. In humans, prolonged exposure to manganese causes neurotoxicity characterized by Parkinson-like symptoms. Mn^2+^ has been shown to mediate DNA damage possibly through the generation of reactive oxygen species. In a recent publication, we showed that Mn induced oxidative DNA damage and caused lesions in thymines. This study further investigates the mechanisms by which cells process Mn^2+^-mediated DNA damage using the yeast *S. cerevisiae*. The strains most sensitive to Mn^2+^ were those defective in base excision repair, glutathione synthesis, and superoxide dismutase mutants. Mn^2+^ caused a dose-dependent increase in the accumulation of mutations using the *CAN1* and *lys2-10A* mutator assays. The spectrum of *CAN1* mutants indicates that exposure to Mn results in accumulation of base substitutions and frameshift mutations. The sensitivity of cells to Mn^2+^ as well as its mutagenic effect was reduced by N-acetylcysteine, glutathione, and Mg^2+^. These data suggest that Mn^2+^ causes oxidative DNA damage that requires base excision repair for processing and that Mn interferes with polymerase fidelity. The status of base excision repair may provide a biomarker for the sensitivity of individuals to manganese.

## 1. Introduction

Manganese (Mn) is a trace element that has been extensively documented for its varied role in the body's homeostasis. As an essential nutrient, Mn is required for the normal function and development of the brain [1], metabolism of proteins, lipids, and carbohydrates [2–4], and also as a functional unit for many enzymes [3–5]. Therefore, deficiencies that affect fetal development [6] and excess Mn (environmental exposure and/or elevated dietary Mn [7]), can result in disorders and disease.

There is increasing concern for the use of organic compounds containing manganese in industrial settings. In recent years, methylcyclopentadienyl manganese tricarbonyl (MMT) gained approval for use in the United States as an octane enhancing fuel additive used in unleaded automotive gasoline. Exposure to Mn has also increased through occupation and environmental settings. This includes agrochemicals such as the fungicides, maneb and mancozeb, and pesticides in the agriculture and forest industries [8] as well as in the case of miners, smelters, welders, and workers in battery factories [9]. The increase in atmospheric levels could result in potential health risks.

At elevated levels of exposure, Mn has been shown to cause manganism, which is an excess of manganese in the basal ganglia [10]. Manganism is characterized by neurological symptoms resembling the dystonic movement associated with Parkinson's disease (PD) [11–13] and therefore is a risk factor for idiopathic Parkinson's disease (IPD). Although Mn has been studied for years, the mechanism by which it causes neuronal damage is not well understood. Studies suggest that neurotoxicity is not caused by a single factor but that it appears to be regulated by a number of factors including apoptosis, oxidative injury, DNA damage, mitochondrial dysfunction, and neuroinflammation [14–18].

The mutagenicity of Mn has been extensively documented [19]. Mn has been shown to cause damage to DNA in multiple cell-based assays [18, 20], to interfere with the fidelity of DNA replication [21], to activate the DNA damage response [22], to induce mutations in T4 phage replication [23] and yeast mitochondria replication [24, 25], and, inhibit repair factor PARP in human cells [26], albeit not scoring as a direct mutagen in the Ames test [27]. Despite its mutagenicity, Mn is not classified as a carcinogen in humans. The reasons for this discrepancy are still not clear.

Research on manganese toxicity has increased in recent years. However, the mechanisms underlying its multiple toxicities (neurotoxicity, genotoxicity, mutagenicity, etc.) [19] remain a mystery. It is possible that redundant mechanisms of DNA repair exist which are effective to handle the levels of Mn to which cells are exposed.

The goal of the current study is to gain insight into the pathways that are involved in DNA damage/repair that contribute to protecting cells from the toxicity of manganese (Mn). The yeast *S. cerevisiae* was utilized as a model system to study the genotoxic effects of Mn. Yeast has proven to be an excellent eukaryotic model for studying metal, and players identified through genetic studies virtually all have homologues in humans. In our study, we use two well-established mutator assays. The *CAN1* assay was used to measure the induction of forward mutations, and the *lys2-10A* reversion assay was used to assess replication fidelity. Furthermore, this study examines the protective effects of the antioxidants N-acetylcysteine and glutathione, as well as Mg^2+^ on Mn-induced toxicity and mutagenesis.

## 2. Materials and Methods

### 2.1. General Genetic Methods and Strains

Yeast extract/peptone/dextrose (YPD, 1% yeast extract, 2% peptone, 2% dextrose, 2% agar) and synthetic complete (SC, 0.67% yeast nitrogen base without amino acid, 0.087% amino acid mixture, 2% dextrose, 2% agar) media or the corresponding drop-out media were as described in [28, 29]. Homozygous haploid deletion strains library (Parental strain BY4741: MATa his3∆1 leu2∆0 met15∆0 ura3∆0) was obtained from Thermo Scientific (Pittsburgh, PA, USA).

### 2.2. Chemicals

Manganese chloride tetrahydrate (MnCl_2_-4H_2_O), N-acetylcysteine (NAC), glutathione (GSH), canavanine, and yeast media were purchased from Sigma-Aldrich (St. Louis, MO, USA).

### 2.3. Sensitivity of Strains to Mn^2+^ and Effect of NAC and GSH

The concentration of Mn^2+^ for strain exposure was determined experimentally using the wild type parental strain, BY4741. Briefly, single colonies were grown for 16 h on YPD with or without Mn^2+^ at 30°C with shaking. Cells were then washed with and resuspended in sterile water. Serial dilutions were spotted onto YPD and plates were incubated at 30°C. Cell growth was monitored daily and sensitivity was scored after 3 days. Colonies were counted and survival (in percentage) was calculated relative to the untreated control. Each strain was tested using at least five independent colonies for each Mn^2+^ concentration tested. To determine the effect of thiol-based antioxidants, cells were cotreated with Mn^2+^ and N-acetylcysteine (NAC) or glutathione (GSH) at the concentrations indicated in each figure. Survival was calculated as described above.

### 2.4. Mutation Analysis

The effect of Mn^2+^ on the accumulation of mutations was assessed by the *CAN1* forward mutation assay and the *lys2-10A* mutation reversion as previously described [30, 31]. Mutation rates were determined by fluctuation analysis using at least five independent colonies [29, 32]. Each fluctuation test was repeated at least three times. The *CAN1* forward mutation assay relies on the introduction of mutations on the *CAN1* gene which encodes the arginine permease allowing mutant cells to grow on plates containing the toxic arginine analog, canavanine. The *lys2-10A* reversion assay is based on the restoration of the open-reading frame in a mononucleotide run of 10 adenines within the *lys2* allele of strain RDKY3590 (Table 1), allowing mutant cells to grow on plates lacking lysine.

### 2.5. DNA Sequence Analysis

Spectrum analysis was carried out by selecting mutants (Can^*r*^) on selective minimum media drop-out plates containing canavanine [29]. Chromosomal DNA was isolated from the mutants and the relevant region of *CAN1* was amplified by PCR and sequenced [30]. Sequence was carried out at MCLAB (San Francisco, CA, USA). Sequence analysis was carried out using Sequencher (Gene Codes, Ann Arbor, MI, USA).

### 2.6. Statistical Analysis

Data analysis and graphing were performed using the GraphPad Prism 4 software package. Specific analysis for each experiment is indicated in each figure legend. In most cases, the mean of at least three experiments is plotted together with the standard deviation. Differences between mean values and multiple groups were analyzed by one-way analysis of variance (ANOVA). Statistical significance was set at *P* < 0.05.

## 3. Results

### 3.1. Sensitivity of **S. cerevisiae ** Strains to Mn^2+^

**Figure 1:**
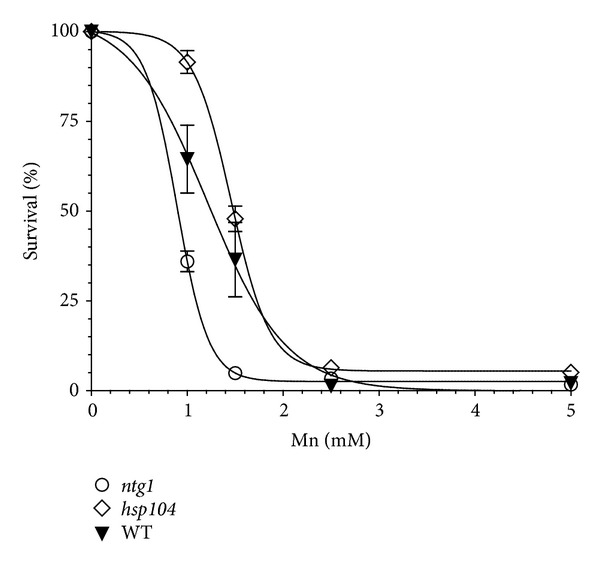
Dose-dependent response of selected yeast strains to Mn^2+^. Wild-type parental strain (BY4741) was tested for growth on media after exposure to 0, 0.5, 1.5, 2.5, and 5 mM Mn^2+^ as indicated in Materials and Methods. Survival was determined by counting the number of colonies in the respective dilutions and calculated based on the growth of cells not exposed to Mn^2+^ (100% survival). Mutant strains *ntg1* and *hsp104* that display increased and reduced sensitivity to Mn^2+^, respectively, are shown for comparison. The curve was fitted by nonlinear Sigmoidal dose response (variable slope).

To perform a comparative analysis of the differential sensitivity of yeast strains, we first determined the dose of Mn^2+^ appropriate for the study. We initially used the wild type strain to determine the range of Mn^2+^ concentrations and found that there was a linear response in a narrow window between 1 and 2.5 mM (Figure 1), with the higher concentration resulting in viability below 5%, which did not significantly increase at higher concentrations of Mn^2+^. All selected strains were then exposed to this range of Mn^2+^ concentrations. Figure 1 shows a comparison between the wild type strain, the disaggregase *hsp104* mutant, which displays higher tolerance to Mn^2+^, and the base excision repair *ntg1* mutant, which is more sensitive. Mn^2+^ at 1.5 mM was determined to be the optimal concentration for the strain comparison (Figure 1). At this concentration, wild-type cells displayed approximately 40% survival and sensitive strains showed higher sensitivity relative to the wild-type strain (Figure 1).

**Figure 2:**
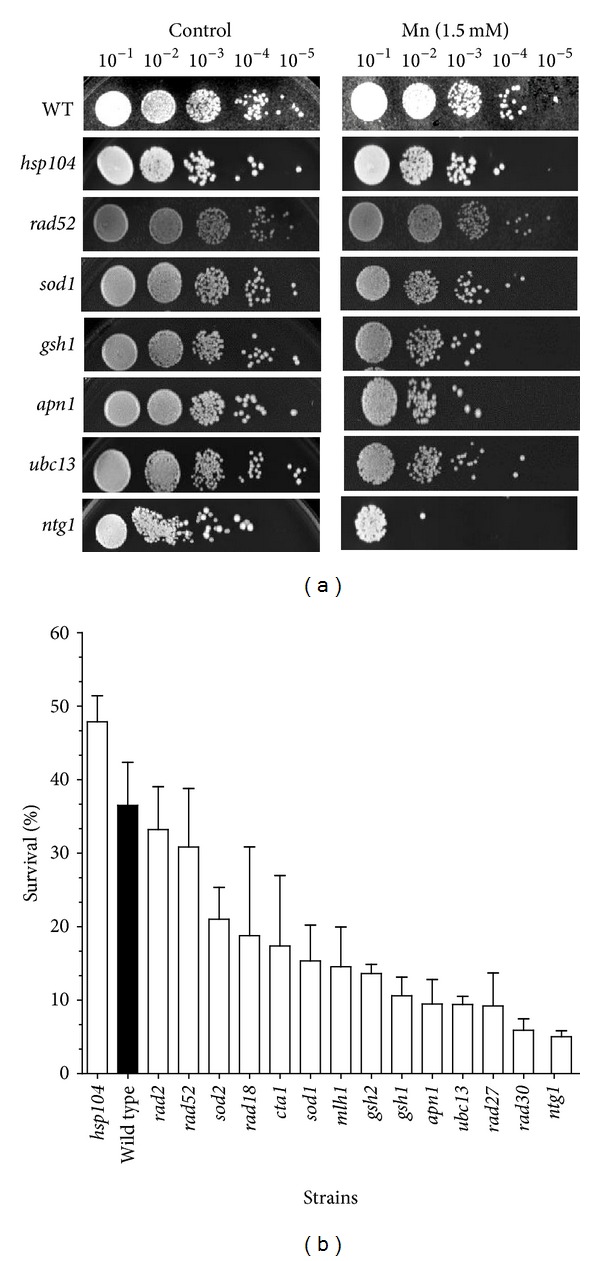
Sensitivity of yeast strains to Mn^2+^. (a) The survival of the strains in 1.5 mM Mn^2+^ was determined as described in Section 2. Serial dilutions (1 : 10–1 : 10^5^) of treated cultures were spotted on YPD plates. Growth was scored after 3 days of incubation at 30°C. The serial dilutions of the strains are shown. (b) Quantification of the survival of the tested strains. Survival was determined by counting the number of colonies in the respective dilutions and calculated based on the growth of strains not treated with Mn^2+^. Strains are presented as being ordered from least to more sensitive. Wild-type strain is depicted by a black bar.

Based on published evidence and a recent report by Stephenson et al. [18], we selected several mutant strains that play a role in the mutagenicity avoidance and may be involved in processing Mn^2+^-induced DNA damage (Table 1). These mutants strains include those defective in nucleotide excision repair (*rad2*), postreplication repair (*rad18*, *rad27a*, and *ubc13*), base excision repair (*apn1*, *rad27*, and *ntg1*), homologous recombination (*rad52*), DNA mismatch repair (*mlh1*), and DNA damage bypass (*rad30*), glutathione synthesis (*gsh1* and *gsh2*), oxidative stress (*sod1*, *sod2*, and *cta1*), and protein disaggregation (*hsp104*). Quantitative analysis involved exposing the cells to Mn^2+^ as described under Materials and Methods and spotting serial dilutions onto nonselective media YPD for colony counting. As observed in Figure 2(a), no significant difference was observed on the growth rate of each strain in the absence of Mn^2+^ (control panel), except for slow growing strain *ntg1*. However, upon treatment with Mn^2+^, the strains displayed differential sensitivity to the metal. All strains tested were sensitive to Mn^2+^ however, only the *hsp104* mutant displayed less sensitivity than the wild type (48% versus 37%; Figure 2(b), black bar). No significant difference between *rad2* (33.2% survival) and the wild type was observed, suggesting that Mn^2+^-induced DNA damage does not result in bulky adducts that require NER for processing. Similarly, no significant difference between *rad52* (31% survival) and the wild type indicates that no significant DNA damage is processed to DNA double-strand breaks that require homologous recombination for repair. Interestingly, the oxidative stress mutants *sod1*, *sod2*, and *cta1*, (15%, 21%, and 17% survival, resp.) were approximately 2-fold more sensitive than wild type and the glutathione synthesis mutants *gsh1* and *gsh2* (10.6% and 13.6% survival, resp.) were 3-fold more sensitive. Mismatch repair mutants *mlh1* displayed 14.5% survival, suggesting that Mn^2+^ induces an increased load of mismatches that cannot be repaired. More striking was the sensitivity of the base excision repair mutants *apn1*, *rad27*, and *ntg1*,*  *(9.5%, 9.2%, and 4.9% survival), which were over 4-fold more sensitive to Mn^2+^ than wild type (Figure 2(b)), with *ntg1* being the most sensitive (7.5-fold). In addition, *ubc13* and *rad30* mutants were also highly sensitive (~4-fold higher than wild type), further suggesting the generation of Mn^2+^-induced DNA damage.

### 3.2. Attenuation of the Sensitivity to Mn^2+^ by Exogenous Antioxidants

**Figure 3:**
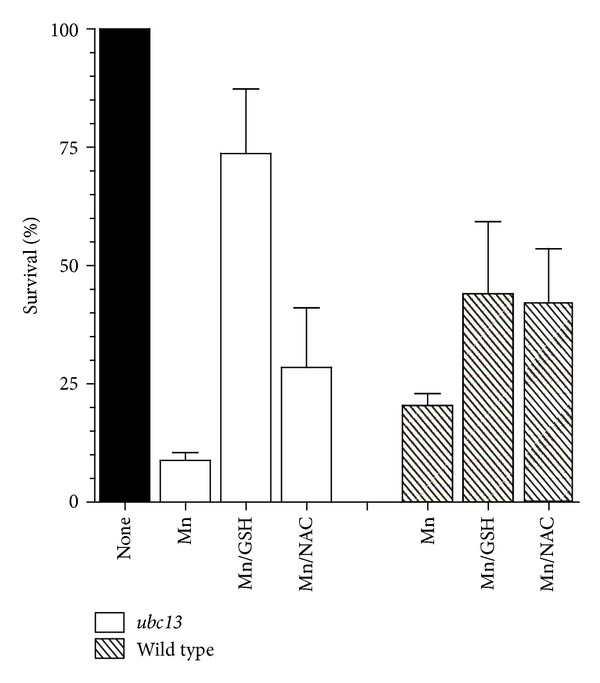
Attenuation of the cytotoxic effect of Mn^2+^ by exogenous antioxidants. Sensitive strain *ubc13* was treated with 1.5 mM Mn^2+^, 1.5 mM Mn^2+^ plus 10 mM glutathione (GSH), and 1.5 mM Mn^2+^ plus 20 mM N-acetylcysteine (NAC), as described in Section 2. Survival was determined relative to untreated strain (100% survival). Wild-type strain was treated with 2 mM Mn^2+^, with or without cotreatment with GSH and NAC, as described in Section 2. At least 5 independent colonies were tested. Average survival plus standard deviation is shown.

Considering that oxidative stress mutants *sod1*, *sod2*, and *cta1* and glutathione synthesis mutants *gsh1* and *ghs2* displayed higher sensitivity to Mn^2+^ than wild-type, we tested if antioxidants would protect from Mn^2+^-induced cytotoxicity. As shown in Figure 3, exogenously added NAC and GSH protected both the wild-type and the hypersensitive strain *ubc13*. The concentration of Mn^2+^ was increased to 2 mM to effectively determine the protective effects of the antioxidants on wild-type cells, resulting in 20% survival. Cotreatment of wild-type cells with 2 mM Mn^2+^ and 20 mM NAC increased the survival to 42%, a 2-fold increase (Figure 3). Similarly, cotreatment with 10 mM GSH increased survival to 44%, a 2-fold increase (Figure 3). To test the protective effect of NAC and GSH on a sensitive strain, we selected *ubc13*, which displayed 9% survival when treated with 1.5 mM Mn^2+^. Cotreatment with 20 mM NAC and 1.5 mM Mn^2+^ increased its survival to 28.5%, a 3-fold increase. Cotreatment with 1.5 mM Mn^2+^ and 10 mM GSH resulted in 74% survival, an 8.4-fold increase (Figure 3). It should be noted that cotreatment with either NAC or GSH alone did not have an effect on the growth of *ubc13* or wild-type strains.

### 3.3. Analysis of the Mn^2+^-Induced Mutator Phenotype of Yeast

**Figure 4:**
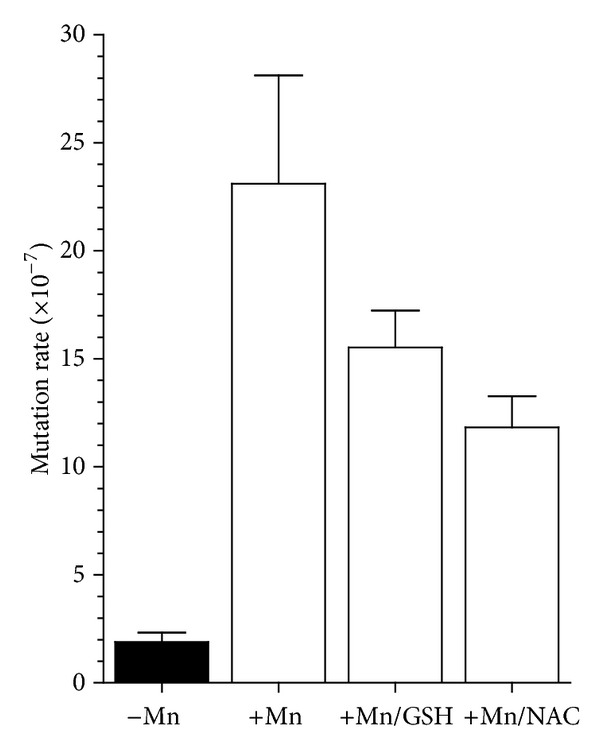
Effect of Mn^2+^ on the mutation rate of the *CAN1* forward mutation assay. The *CAN1* assay detects any mutation which inactivates the *CAN1* gene (arginine permease) and allows cells to grow on plates containing the toxic arginine analog, canavanine. The assay was performed using the wild-type strain in the presence of 1.5 mM Mn^2+^ or cotreated with 1.5 mM Mn^2+^ and 10 mM GSH or 1.5 mM Mn^2+^ and 10 mM NAC as indicated. Appearance of colonies on canavanine containing plates is scored and mutation rates are determined as described in Section 2. and standard deviation is included at the top of each bar.

The mutagenicity of Mn^2+^ has been extensively documented [19]. To determine the extent to which exposure to Mn^2+^ increases the accumulations of mutations and to quantify the increase in the mutation rate of wild-type yeast cells, we utilized the *CAN1* forward mutation assay [33], as described in Section 2. As shown in Figure 4, the mutation rate increased 12-fold (from 1.9 × 10^−7^ to 23.1 × 10^−7^) when wild-type cells were treated with 1.5 mM Mn^2+^. Based on the ability of antioxidants to reduce the toxicity of Mn^2+^ (Figure 3), we tested if cotreatment with NAC or GSH could also reduce the Mn^2+^-induced increase of the mutation rate. In fact, 20 mM NAC reduced the mutation rate by 1.5-fold (from 23.1 × 10^−7^ to 15.5 × 10^−7^), while 10 mM GSH reduced the mutation rate by 2-fold (from 23.1 × 10^−7^ to 11.8 × 10^−7^), consistent with the ability of these antioxidants to reduce Mn-induced toxicity (Figure 3).

### 3.4. Mutation Spectrum of CAN-Resistant Mutants

The *CAN1* forward mutations assay provides a useful tool to identify the nature of the mutations that are generated from Mn^2+^ exposure. For this purpose, we amplified the *CAN1* gene from canavanine-resistant colonies treated with 1.5 mM Mn^2+^ and completely sequenced the ORF to identify the mutation.  Table 2 shows the spectrum of mutations of 20 independent canavanine-resistant colonies. A single mutation was identified in each isolate. Mutations are indicated first by the original base, its numerical sequence position, followed by the mutant base. The majority (70%) of the mutations were base-substitution mutations with 40% (8/20) being transitions and 30% transversions (6/20). The rest (30%) were frameshift mutations, of which 10% (2/20) were insertions and 20% (4/20) were deletions of single nucleotides at the position indicated. No complex mutations such as large deletions, insertions, duplications, or gross chromosomal rearrangements were found. No hotspot was found, although some base-substitution mutations were observed twice (G1196A, G1555A and A1417T; Table 2).

### 3.5. Mn^2+^-Induced Reversion Mutations in the **lys2-10A ** Allele Which Can Be Reduced by Mg^2+^

**Figure 5:**
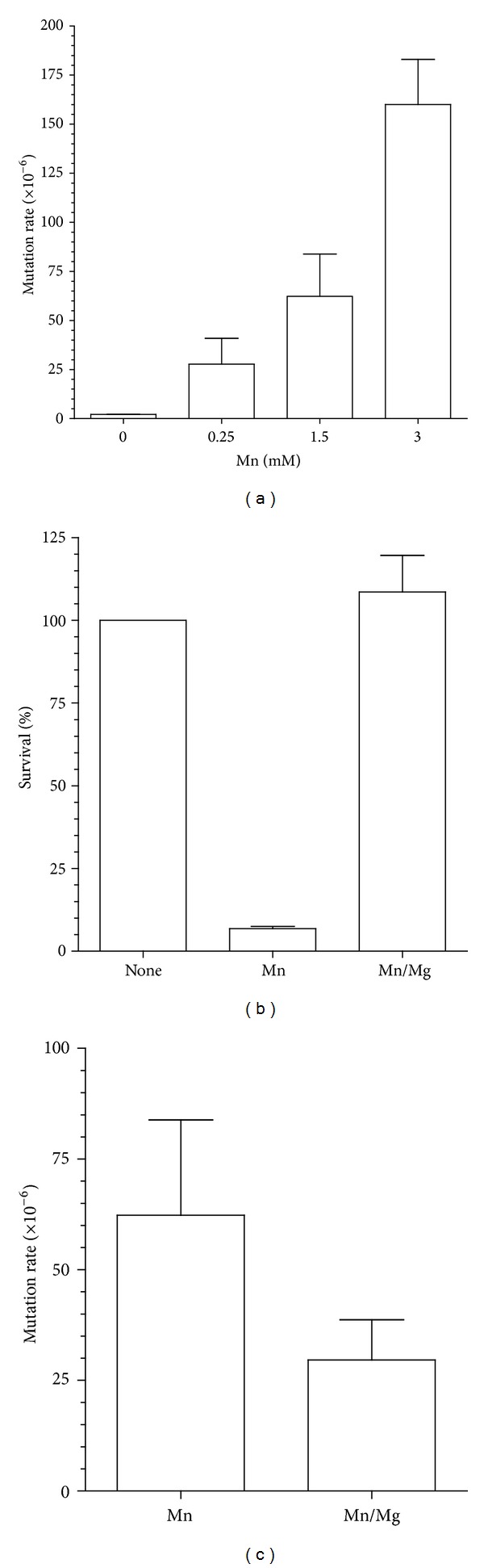
Effect of Mn^2+^ and Mg^2+^ on the mutation rate of the reversion of the *lys2-10A* allele. (a) Mutation rates determination by the yeast mutator assay using a strain carrying the *lys2-10A* allele was performed in the presence of increasing concentrations of Mn^2+^. The appearance of Lys^+^ revertant colonies indicates a mutator phenotype. Rates are calculated as described in Section 2 and standard deviation is included at the top of each bar. (b) Cotreatment with 10 mM Mg^2+^ protects cells from the toxicity of Mn^2+^. Survival was determined as described in Section 2. (c) Cotreatment with 10 mM Mg^2+^ reduces the accumulation of mutations on the *lys2-10A* allele induced by 1.5 mM Mn^2+^. Each bar corresponds to the average of three sets of experiments using five independent colonies per set.

The Mn^2+^-induced accumulation of frameshift mutations prompted us to investigate if Mn^2+^ may be promoting polymerase slippage. For this purpose, we treated a yeast strain carrying the *lys2-10A* allele, where the *LYS2* gene has a mononucleotide run of 10 adenines resulting in an out-of-frame gene, which can be restored by a frameshift mutation. We observed a dose-dependent increase in the mutation rate of this strain with increasing concentrations of Mn^2+^ (Figure 5(a)). Even at low concentrations of Mn^2+^ (0.25 mM), the mutation rate increased by 13-fold (from 2.1 × 10^−6^ to 27.8 × 10^−6^) and was 30-fold (2.1 × 10^−6^ to 62.3 × 10^−6^) and 76-fold (2.1 × 10^−6^ to 160 × 10^−6^) higher at 1.5 mM and 3 mM concentrations of Mn^2+^, respectively (Figure 5(a)). To determine if Mn^2+^ was displacing Mg^2+^ in the DNA synthesis reaction, we tested if exogenously added Mg^2+^ could both increase the survival of the strain as well as reduce its mutator phenotype. As shown in Figure 5(b), cotreatment of the strain with 1.5 mM Mn^2+^ and 10 mM Mg^2+^ significantly increased the survival to 100% (figure) and reduced the mutation rate by 2-fold (Figure 5(c)). 

## 4. Discussion

Manganese is an essential trace metal required for normal physiological function. However, excess Mn exposure is associated with several disease states. Significant research focuses on chronic exposure to Mn which has been shown to cause manganism [10], a neurological disease referred to as idiopathic Parkinson's disease (IPD) that presents symptoms resembling the dystonic movement associated with Parkinson's disease (PD) [11–13]. Numerous studies suggest that the neurotoxicity as a result of Mn exposure is a consequence of a variety of factors including apoptosis, oxidative injury, DNA damage, mitochondrial dysfunction, and neuroinflammation [14–18]. Of particular interest is the mutagenicity of Mn. Despite extensive knowledge of the DNA damaging properties of Mn, little is known about the pathways involved in the response and repair of Mn-induced DNA damage.

In the present work, we investigate the contribution of various DNA repair pathways to the survival of yeast cells exposed to Mn toxicity. We selected mutant strains in key components of the major DNA repair pathways, as described in Table 2. Initial observation indicates that yeast cells are relatively tolerant to Mn^2+^, displaying reduced viability only when concentrations reach over 1 mM (Figure 2), which is several orders of magnitude higher than for other metals, such as Cd^2+^, which is toxic at the *μ*M level [31]. Interestingly, the sensitivity of yeast cells to Mn^2+^ is almost complete when the concentration of Mn^2+^ reaches 2.5 mM (Figure 2) displaying a linear response within this concentration window. For this reason, the strain comparison was performed at the 1.5 mM concentration. All strains displayed varying degrees of sensitivity, and all except the *hsp104* strain, were more sensitive than the wild type, suggesting that no significant toxic levels of protein aggregation are induced by Mn^2+^.

Cells possess three major excision repair pathways: (i) base excision repair (BER) which is responsible for the repair of damaged bases resulting primarily from oxidative damage [34], (ii) nucleotide excision repair (NER) which plays a major role in the repair of large DNA adducts and UV damaged DNA [35, 36], and (iii) DNA mismatch repair (MMR), a postreplicative mechanism, improves the fidelity of DNA replication by removing misincorporated bases by the DNA polymerase [37]. In addition, cells possess recombination repair, which in yeast is primarily performed by homologous recombination (HR) [38]. These pathways act in concert to respond to exogenous damage and guarantee genome stability. Some of these pathways have been shown to be defective in neurodegenerative diseases [39, 40] and participate in response to neurotoxic agents [41, 42]. Our data suggests that BER plays a major role in the cellular response to toxic levels of Mn^2+^ as mutants *apn1*, *rad27*, and *ntg1* were more than 4-fold sensitive to Mn^2+^ than wild type (Figure 2(b)) and *ntg1* was the most sensitive (7.5-fold). *NTG1* is a DNA N-glycosylase which removes the oxidized damaged base on both nuclear and mitochondrial DNA [43]. The DNA damage generated by Mn^2+^ appears to interfere with DNA replication, as indicated by the high sensitivity of strains *ubc13*, a DNA-damage-inducible gene, member of the error-free postreplication repair pathway [44], and *rad30* mutants, which are defective in translesion synthesis DNA polymerase eta, required for bypass synthesis at sites where replication forks are stalled due to damaged bases. Conversely, NER does not appear to play a major role in the repair of Mn^2+^-induced DNA damage, as indicated by similar survival of *rad2* mutant to the wild type. Similarly, the lack of a strong Mn^2+^-induced phenotype in the *rad52* strain suggests that no significant DNA damage is processed to DNA double-strand breaks, which requires homologous recombination for repair.

It appears that oxidative stress plays a major role in Mn^2+^ cytotoxicity as indicated by the increased sensitivity of the superoxide dismutase (*sod1* and* sod2*) and catalase mutants (*cta1*). This is further supported by the ability of NAC to improve the survival of the wild-type strain and the DNA repair strain *ubc13* (Figure 3). Exogenous addition of glutathione, which serves both as a reducing agent and a chelator to Mn, further protected the strains from Mn^2+^ exposure.

A significant increase in the accumulation of mutations was observed in cells exposed to Mn^2+^, using two distinct mutator assays. The *CAN1* forward mutation assay indicated a 12-fold increase in the mutation rate when cells were exposed to 1.5 mM Mn^2+^. Similar to the effect on survival, NAC and GSH reduced the increase in the mutation rate, suggesting that the mutations are at least the result of oxidative damage to DNA. Analysis of the mutations in the *CAN1* gene in these yeast cells indicates that most base substitutions are accumulated (70%), while 30% were frameshifts mutations. In combination with the increased mutation rate, cells exposed to Mn^2+^ have a significantly higher accumulation of frame shift mutations. This is distinct from spontaneous mutations (not exposed to Mn^2+^), where 10% of the mutants analyzed had complex mutations [29, 45]. The increase in frameshift mutations was also observed when the mutation rate was measured using the *lys2-10A* allele. This increase was dose-dependent and ameliorated by Mg^2+^, concomitant with an increase in cell survival. In fact, Mg^2+^ has been shown to protect cells from Mn^2+^ toxicity [46–48]. It is possible that the mutation rate increase is the result of Mn^2+^ intoxication of the DNA polymerase by displacing Mg^2+^ [21], which would require MMR for repair, explaining the increased sensitivity of the *mlh1* strain.

The adverse effect of Mn^2+^ in DNA polymerase fidelity has been previously reported [21] and proposed to be due to replacement of Mg^2+^, which is essential in the reaction. However, recently, a series of studies have shown that some viral polymerases, such as those of coronavirus [49] and poliovirus [50], have exclusive requirement for Mn^+2^ in their synthetic activity. Similarly, the incorporation of nonnucleoside triphosphate analogs is accomplished by X family DNA polymerases in an Mn-dependent manner [51], while cellular error-prone DNA polymerase iota, isolated from tumor cells, was shown to utilize Mn^2+^ [52] in DNA synthesis. This is an interesting observation because DNA polymerase iota is inducible by Mn^2+^ and could in part contribute to the mutagenesis observed in Mn^2+^ exposed cells.

Most published work on the toxicity of manganese has focused on Mn^2+^, while there was some claim that Mn^3+^ was the toxic species. However, recent work indicates that Mn^3+^ has a significantly reduced toxicity compared to Mn^2+^ [53, 54]. In addition, since manganese has a similar ionic radius to calcium, Mn^2+^ has been shown to interfere with Ca^2+^ metabolism [19, 55]. However, there are no reports of Ca^2+^ having an effect on BER.

The data presented in this study indicates that Mn^2+^-induced DNA damage is in part due to oxidative stress and requires base excision repair. Considering the well-known relationship between DNA repair defects and neurodegenerative diseases, and the involvement of DNA repair in response to neurotoxic agents, the status of base excision repair, or some of its key components, may prove to be useful as biomarkers to determine the susceptibility to toxic damage from excess exposure to Mn^2+^. There is currently a lack of well-validated biomarkers for manganese exposure. Manganese overexposure leads to cognitive, motor, behavioral effects in children [56] and manganese is associated with Parkinson's disease in adults [11–13]. Persons most likely to be exposed to excessive levels of manganese are manganese coal miners and welders. However, there is currently no way to determine who will suffer severe effects after Mn overexposure. Thus, preventive strategies and biomarker development for BER status are strongly supported by our findings. An assay that monitors the BER status of exposed individuals could be used in conjunction with other recently proposed biomarkers for Mn exposure which measure delta-amino levulinic acid levels [57] and the Mn/Fe ratio [58]. While these two biomarkers can detect exposure to Mn, an assay evaluating BER status would be of more value as a preventative strategy with its inherent potential to distinguish individuals who would be more severely affected by Mn exposure from those who would not.

## Figures and Tables

**Table 1:** Strains used in this study.

Gene	ORF	Function
*HSP104 *	YLL026W	Protein disaggregase
*RAD2 *	YGR258C	Nucleotide excision repair endonuclease
*RAD52 *	YML032C	Homologous recombination
*SOD2 *	YHR008C	Mitochondrial superoxide dismutase
*RAD18 *	YCR066W	Postreplication repair
*CTA1 *	YDR256C	Catalase activity
*SOD1 *	YJR104C	Superoxide dismutase activity
*MLH1 *	YMR167W	Mismatch repair
*GSH2 *	YOL049W	Glutathione synthetase activity
*GSH1 *	YJL101C	Glutamate-cysteine ligase activity
*APN1 *	YKL114C	Base excision repair
*UBC13 *	YDR092W	DNA postreplication repair
*RAD27 *	YKL113C	Base excision repair, DNA replication
*RAD30 *	YDR419W	Bypass synthesis DNA polymerase
*NTG1 *	YAL015C	Base excision repair

Wild type: strain BY4741 (MATa his3Δ1 leu2Δ0 met15Δ0 ura3Δ0).

RDKY3590 (MATa, ura3-52, leu2D1, trp1D63, hom3-10; lys210A).

**Table 2:** *CAN1* mutation spectrum of wild-type yeast exposed to Mn^2+^.

Base substitution mutations	
Transitions (8/20)	Transversions (6/20)
G522A G550A C623T G670A G1196A(×2) G1555A (×2)	A312T A375C T380G A1417T (×2) A1645T

Frameshift mutations	
Insertions (2/20)	Deletions (4/20)

T740 T628	C417 T1143 G1259 G1474
